# Binding site asymmetry in human transthyretin: insights from a joint neutron and X-ray crystallographic analysis using perdeuterated protein

**DOI:** 10.1107/S2052252514021113

**Published:** 2014-10-21

**Authors:** Melina Haupt, Matthew P. Blakeley, Stuart J. Fisher, Sax A. Mason, Jon B. Cooper, Edward P. Mitchell, V. Trevor Forsyth

**Affiliations:** a Facility of Natural Sciences, Institute of Science and Technology in Medicine, Keele University, Staffordshire ST5 5BG, United Kingdom; b Institut Laue-Langevin, 71, avenue des Martyrs, Grenoble, CS 20156, France; c Partnership for Structural Biology, 71, avenue des Martyrs, Grenoble, CS 20156, France; d Department of Molecular Biology, University of Salzburg, Billrothstrasse 11, Salzburg, 5020, Austria; e Diamond Light Source, Didcot, Oxfordshire OX11 0DE, United Kingdom; f Division of Medicine (Royal Free Campus), University College London, Rowland Hill Street, London NW3 2PF, United Kingdom; g Business Development Office, European Synchrotron Radiation Facility, Grenoble, 38042, France

**Keywords:** transthyretin, amyloid assembly, neutron crystallography, deuteration

## Abstract

Human transthyretin has an intrinsic tendency to form amyloid fibrils and is heavily implicated in senile systemic amyloidosis. Here, detailed neutron structural studies of perdeuterated transthyretin are described. The analyses, which fully exploit the enhanced visibility of isotopically replaced hydrogen atoms, yield new information on the stability of the protein and the possible mechanisms of amyloid formation. Residue Ser117 may play a pivotal role in that a single water molecule is closely associated with the γ-hydrogen atoms in one of the binding pockets, and could be important in determining which of the two sites is available to the substrate. The hydrogen-bond network at the monomer–monomer interface is more extensive than that at the dimer–dimer interface. Additionally, the edge strands of the primary dimer are seen to be favourable for continuation of the β-sheet and the formation of an extended cross-β structure through sequential dimer couplings. It is argued that the precursor to fibril formation is the dimeric form of the protein.

## Abstract 2

A neutron crystallographic study of perdeuterated transthyretin reveals important aspects of the structure relating to its stability and its propensity to form fibrils, as well as evidence of a single water molecule that affects the symmetry of the two binding pockets.

## 1. Introduction   

Human transthyretin (TTR) is a 55 kDa homotetramer that transports thyroxine and retinol-binding protein in the blood and cerebrospinal fluid (Hamilton & Benson, 2001 ▶; Schreiber *et al.*, 1995 ▶). Wild-type TTR is inherently amyloidogenic and can lead to senile systemic amyloidosis (SSA). SSA is the most widespread form of TTR amyloid disease and is associated with microscopic amyloid deposits of uncertain clinical significance in many organs of all individuals who are over 80 years old, 25% of whom develop disease symptoms (Westermark *et al.*, 1990 ▶). Point mutations in the TTR gene often result in early onset of disease, presumably due to destabilization of the tetramer, rendering the protein prone to dissociation and aggregation. This hereditary and often fatal form of amyloidosis is also referred to as familial amyloid polyneuropathy (Planté-Bordeneuve & Said, 2011 ▶). More than 100 TTR mutations have been described of which many are pathogenic (Connors *et al.*, 2003 ▶). A regularly updated list of mutations together with their primary citations can be found on the website http://amyloidosismutations.com/mut-attr.php. A promising approach to the treatment of these diseases is the stabilization of the native fold using drugs that inhibit dissociation of the TTR tetramer and the subsequent aggregation of the protein into its fibrillar form (Connelly *et al.*, 2010 ▶; Kolstoe *et al.*, 2010 ▶; Sekijima *et al.*, 2008 ▶). Several drug candidates have been identified and tested and some have been shown to suspend polyneuropathy by raising the kinetic barrier of tetramer dissociation (Choi *et al.*, 2010 ▶; Sekijima *et al.*, 2008 ▶). Other approaches have resulted in the development of a potent and selective class of bivalent palindromic ligands (Green *et al.*, 2003 ▶; Kolstoe *et al.*, 2010 ▶).

The stability of the homotetramer of wild-type TTR and the changes associated with point mutations or ageing processes are believed to be closely linked to the electrostatic and hydrogen-bonding interactions present at the protein–protein interface. Interestingly, wild-type TTR does not necessarily degrade prior to aggregation and deposition in tissue (Ihse *et al.*, 2008 ▶; Pepys, 2009 ▶). It has been shown that amyloid fibrils contain a high percentage of wild-type TTR for individuals heterozygous for the Val30Met variant (Ihse *et al.*, 2011 ▶; Tsuchiya-Suzuki *et al.*, 2011 ▶).

Neutron diffraction is a powerful tool for structural analysis which strongly complements X-ray techniques because it enables key details of hydration and protonation to be revealed (Forsyth *et al.*, 1989 ▶; Langan *et al.*, 1992 ▶; Kovalevsky *et al.*, 2010 ▶; Tomanicek *et al.*, 2010 ▶, 2011 ▶; Howard *et al.*, 2011 ▶; Kovalevsky *et al.*, 2011 ▶; Weber *et al.*, 2013 ▶; Cuypers, Mason *et al.*, 2013 ▶; Casadei *et al.*, 2014 ▶; Langan *et al.*, 2014 ▶). This occurs because, in contrast to the situation with X-ray diffraction, hydrogen and its isotope deuterium have scattering powers for neutrons that are comparable with those for the other atoms commonly found in biological macromolecules, and are therefore more easily visible in neutron diffraction studies (Blakeley, 2009 ▶). Perdeuteration of the sample, in which all hydrogen atoms are replaced by deuterium, facilitates data collection from smaller samples and those with larger unit cells (Hazemann *et al.*, 2005 ▶; Petit-Haertlein *et al.*, 2009 ▶), and is invaluable to other techniques used in structural biology and soft matter research including small-angle neutron scattering (Cuypers, Trubitsyna *et al.*, 2013 ▶), neutron reflectometry (Grage *et al.*, 2011 ▶), fibre diffraction (Shotton *et al.*, 1997 ▶; Gardner *et al.*, 2004 ▶; Nishiyama *et al.*, 2010 ▶) and NMR (Varga *et al.*, 2007 ▶).

A recent review analysing the output of almost 200 X-ray crystal structures of transthyretin, its mutants, complexes and related proteins concludes that despite the abundance of data available, no consistent and conclusive model for mutation-induced structural changes or for the mechanism of amyloid formation has been put forward (Palaninathan, 2012 ▶). The neutron crystallographic analysis presented here sheds light on a vitally important aspect – the role of hydrogen-atom interactions in the stability of the structure. In addition to the biological interest of these results, it is noted that the study was carried out using two datasets recorded from completely different neutron diffractometers at the Institut Laue-Langevin (ILL). One was a quasi-Laue machine dedicated to neutron protein crystallography, and the other was a monochromatic instrument that is typically used for studies in structural chemistry, small proteins, as well as fibre diffraction studies of biological and synthetic polymers. Hence the results, which are closely consistent, are of technical interest for an understanding of the complementary scope of each approach.

## 2. Experimental procedures   

### 2.1. Sample preparation   

Human TTR was prepared and crystallized as described previously (Haupt *et al.*, 2011 ▶). This involved the use of a pET-M11 bacterial expression vector with an N-terminal 6His tag and a TEV cleavage site (EMBL Protein and Purification Facility, Germany) with the TTR gene ligated *via* NcoI and Acc65I restriction sites. Following transformation into BL21(DE3) cells (Invitrogen), the cells were adapted to perdeuterated ENFORS minimal medium and grown in a fed batch fermentation process using U-D8-glycerol (99% deuterium, Euriso-top) as the carbon source. Purification was carried out using an Ni^2+^ column and subsequent gel filtration. MALDI–TOF mass spectrometry showed deuteration of 100% of all non-exchangeable hydrogen atoms. Large single crystals of ≥3 mm^3^ were grown using the vapour diffusion method in sitting drops in 2.15 *M* malonate buffer pD 6.0 and 23 mg ml^−1^ protein concentration.

### 2.2. Quasi-Laue neutron data collection (LADI-III)   

Details of the quasi-Laue neutron diffraction data collection have been reported previously (Haupt *et al.*, 2011 ▶). Neutron quasi-Laue diffraction data were collected at room temperature on the LADI-III instrument (Blakeley *et al.*, 2010 ▶) installed on cold neutron guide H142 at the Institut Laue-Langevin (ILL). Using a crystal of perdeuterated TTR with volume of ∼3.4 mm^3^, diffraction data were collected to 2.0 Å resolution. As is typical for a Laue crystallography experiment, the crystal was held stationary at a different ϕ (vertical rotation axis) setting for each exposure. Initially, 12 contiguous images (Δϕ = 7°) were collected using an exposure time of 8 h per image in order to collect the high-resolution data, followed by a low-resolution pass of 11 contiguous images (Δϕ = 7°) using an exposure time of 2 h per image. Thus, the complete dataset comprised 23 images with an average exposure time of 5.13 h per image. The neutron diffraction images were processed using the *LAUE* suite program *LAUEGEN* (Campbell, 1995 ▶; Helliwell *et al.*, 1989 ▶), which was modified to account for the cylindrical geometry of the detector (Campbell *et al.*, 1998 ▶). The program *LSCALE* (Arzt, 1999 ▶) was used to determine the wavelength-normalization curve using the intensities of symmetry-equivalent reflections measured at different wavelengths and to apply wavelength-normalization calculations to the observed data. The data were then scaled and merged in *SCALA* (Evans, 2006 ▶). Relevant data collection statistics are summarized in Table 1 ▶.

### 2.3. Thermal neutron data collection (D19)   

Monochromatic data were recorded at room temperature using the D19 diffractometer at the ILL. Data were recorded to a resolution of 2.3 Å using ω-step scans of 0.07°, to preset monitor counts, with typical times per frame of 87 or 173 s. The wavelength used was 2.42 Å produced using a graphite monochromator. The λ/2 harmonic was reduced below 1% using a graphite filter ahead of the sample position. The detector was a large, curved multiwire proportional counter having an angular coverage of 120° in the horizontal plane and 30° vertically. The data were processed using the ILL program *RETREAT* (Wilkinson *et al.*, 1988 ▶). The use of perdeuterated protein effectively obviated the need for an absorption correction. Data were merged using *SCALA* from the *CCP*4 suite (Winn *et al.*, 2011 ▶). A summary of the experimental and refinement statistics is given in Table 1 ▶.

### 2.4. X-ray data collection and structure refinement   

X-ray diffraction data were recorded at room temperature using beamline ID23-1 (Flot *et al.*, 2010 ▶) at the European Synchrotron Radiation Facility (ESRF), using a heavily attenuated X-ray beam (0.74%) of wavelength 0.9791 Å. Integrated and scaled reflection intensities were obtained using *MOSFLM* (Battye *et al.*, 2011 ▶; Leslie, 2006 ▶) and *SCALA*, and converted to structure factors using *TRUNCATE* (Winn *et al.*, 2011 ▶). Radiation-damage effects were monitored using the inter-frame merging *R* factors and *B* factors.

PDB code 3ipe was used as the starting model. All water molecules and multiple conformations were removed and the model was randomly perturbed to reduce model bias. Initially, rigid-body refinement was then conducted using *PHENIX* (Adams *et al.*, 2010 ▶) to 1.95 Å. Following this a series of restrained refinements were conducted and the resulting σ_A_ weighted *F*_o_ − *F*_c_ electron-density map contoured to 3σ was used to model 148 solvent molecules and a number of multiple conformations. Further rounds of restrained refinement were conducted. The refinement converged with a final *R* factor of 15.2% and *R*_free_ of 20.1%. Full refinement statistics are shown in Table 1 ▶ and the model has been deposited in the Protein Data Bank (http://www.rcsb.org/pdb) under accession code 4pvl.

### 2.5. Joint neutron and X-ray structural refinement   

The refined X-ray model of perdeuterated TTR to 1.95 Å resolution determined at room temperature was used as the starting model for the joint X-ray and neutron refinement using the *phenix.refine* program (Afonine *et al.*, 2010 ▶) in the *PHENIX* package (version 1.6.2). The model was first modified by removing all water molecules, resetting the atomic displacement parameters (ADPs) of all atoms to 25 Å^2^, and randomly perturbing the atomic coordinates to reduce model bias. Deuterium atoms were then added to the protein model using the *ReadySet* option in *PHENIX* (Adams *et al.*, 2010 ▶).

Initially rigid-body refinement was performed, followed by several cycles of maximum-likelihood-based refinement of individual coordinates, isotropic ADPs and atomic occupancies of side-chain multiple conformations. Using the modelling program *Coot* (Emsley *et al.*, 2010 ▶), rotamer- and torsion-angle adjustments were made manually throughout the model according to positive nuclear scattering density in both σ_A_-weighted 2*F*_o_ − *F*_c_ and *F*_o_ − *F*_c_ maps. The first nine residues at the N-terminus, along with the last two residues at the C-terminus of both chains A and B were found to be disordered in the X-ray and neutron scattering density maps and thus were not modelled, with the exception of Lys-9 which could be modelled with the neutron data in chain B. Molecules of D_2_O were added to the model according to positive neutron scattering length density in σ_A_-weighted *F*_o_ − *F*_c_ maps, with manual adjustment of all D_2_O molecules completed using both σ_A_-weighted 2*F*_o_ − *F*_c_ and *F*_o_ − *F*_c_ maps. A total of 89 solvent molecules were included in the final round of refinement using *phenix.refine* for the Laue dataset, and 84 in the case of the monochromatic dataset. Based on the density maps, these were modelled as full D_2_O molecules, D—O or ‘O-only’ (*i.e* exhibiting spherical density); a summary is given in Table 1 ▶. A minimal r.m.s. level of 1.5 was used for the attribution of water molecules. The same strategy was applied for both quasi-Laue diffraction and monochromatic data. The quasi-Laue neutron *R*_work_ and *R*_free_ values for the final model were 20.9% and 27.1%, respectively, while the X-ray *R*_work_ and *R*_free_ values were 15.3% and 20.3%, respectively. For monochromatic neutron joint refinement the *R*_work_ and *R*_free_ values for the final model were 20.9% and 26.2% for neutrons and 15.6% and 18.5% for X-rays, respectively. The final refinement statistics from *phenix.refine* are summarized in Table 1 ▶. *MolProbity* (Davis *et al.*, 2007 ▶) was used to analyze the stereochemistry of the final model (see Table 1 ▶). The models and the diffraction data have been deposited in the Protein Data Bank (http://www.rcsb.org/pdb) under accession codes 4pvm and 4pvn for the quasi-Laue and monochromatic neutron models, respectively.

## 3. Results   

### 3.1. Neutron crystal structures   

As noted above, two independent neutron datasets were obtained from the same crystal using two different diffractometers at the ILL. The first was recorded using the quasi-Laue diffractometer LADI-III (to a resolution of 2.0 Å), and the second was recorded using the monochromatic thermal neutron diffractometer D19 (to a resolution of 2.3 Å). Both datasets were recorded at room temperature. An X-ray dataset was subsequently collected to 1.95 Å resolution at room temperature from a crystal originating from the same crystallization well. The availability of both X-ray and neutron diffraction data allowed joint refinements, increasing the data-to-parameter ratio and reducing the influence of systematic errors. A summary of the quasi-Laue neutron, monochromatic neutron, and X-ray data collection statistics is given in Table 1 ▶, along with details of each of the refinements carried out. A comparison of the two neutron-derived structures showed close agreement; a superposition was carried out using *Coot* (Emsley *et al.*, 2010 ▶) and the mean deviation was calculated to be 0.13 Å. It is interesting to note that while the study was based on the use of perdeuterated protein (as assessed by mass spectrometry), small amounts of exchange of D back to H can be expected. This may happen during purification (where hydrogenated reagents are usually used), and may not fully reverse during crystallization in fully deuterated buffers. Such back-exchange typically involved amide backbone hydrogen atoms and was identified during the respective structure refinements. The results described here for perdeuterated wild-type TTR have been compared with the crystallographic study of a truncated form of the hydrogenated protein (Yokoyama *et al.*, 2012 ▶). This study was carried out to a resolution of 2 Å using the iBIX instrument at J-PARC (Tanaka *et al.*, 2010 ▶). The comparison shows good consistency between the two structures, and demonstrates the major advantages arising from the use of perdeuterated protein. As has been noted previously, the negative scattering length of hydrogen for neutrons has a number of important implications for neutron macromolecular crystallography. In addition to hydrogen incoherent scattering and absorption issues that affect data precision and data correction, the character of neutron scattering length density maps obtained are very different. If, as is often the case in protein crystallography, high-resolution data are not obtained, cancellation can occur between positive and negative regions of density maps, and this may limit the clarity and interpretation of the analysis. Additionally, given the significantly larger scattering power of deuterium by comparison with its hydrogen isotope, it is evident that deuterium atoms will be more easily identifiable, even at lower resolutions. In the sections that follow, we note points of comparison between this study and that of Yokoyama *et al.* (2012 ▶).

### 3.2. Tertiary and quaternary structure   

**Figure 1:**
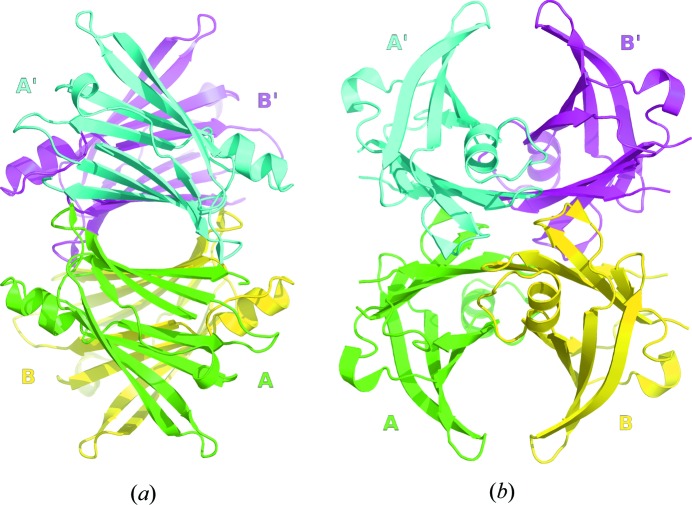
Front view along the bivalent hormone binding channel (*a*) and side view perpendicular to the binding channel (*b*) of human transthyretin. Subunit A (green) forms a continuous saddle-like curved β-sheet with subunit B (yellow). The concave shaped β-sheet saddle constitutes the dimer–dimer interface, leaving a spacious channel. Subunits A and A′ (cyan) constitute the hormone binding site A, and subunits B and B′ (magenta) enclose binding site B.

**Figure 2:**
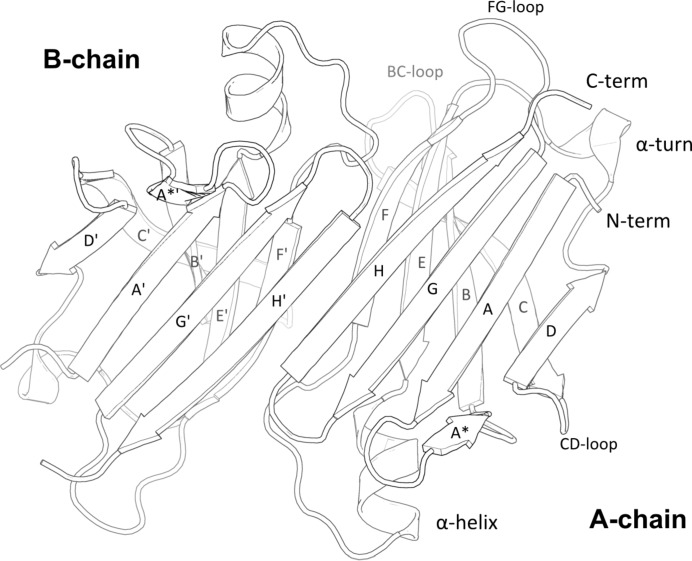
Schematic representation of the primary dimer. The dimer–dimer interface is oriented towards the observer.

Native human TTR is known to be a homotetramer, or, more precisely, a dimer of dimers (Fig. 1 ▶). The monomer consists of two four-stranded β-sheets, arranged in a sandwich-like tertiary structure in which β-strands HGAD are opposed to β-strands FEBC (Fig. 2 ▶). There is a short β-strand, A*, that is folded back relative to strand A *via* a π-turn (*i*+5) and which is involved in the dimer–dimer contact. The only helical part of the secondary structure is an α-helix located between strands E and F, and also one solvent-exposed β-turn (Thr60–Phe64) that mediates a crystal contact.

**Figure 3:**
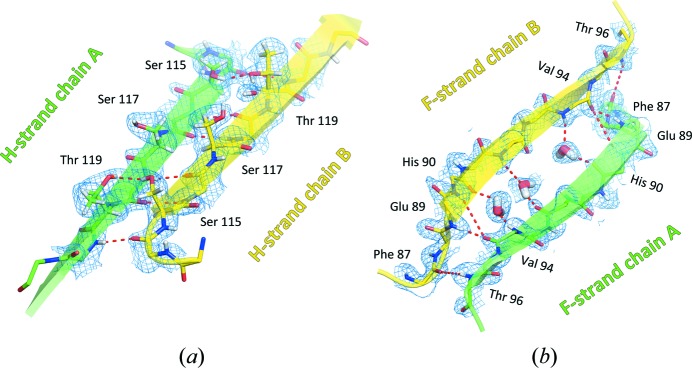
The monomer–monomer interface. (*a*) The continuous β-sheet formed by the monomers A (green) and B (yellow), as seen from the binding channel. (*b*) The somewhat looser assembly on the solvent-exposed side of the β-sandwich. Three water molecules are wedged between strands F and F′, preventing a smooth circular sequel of the CBEF sheet.

**Figure 4:**
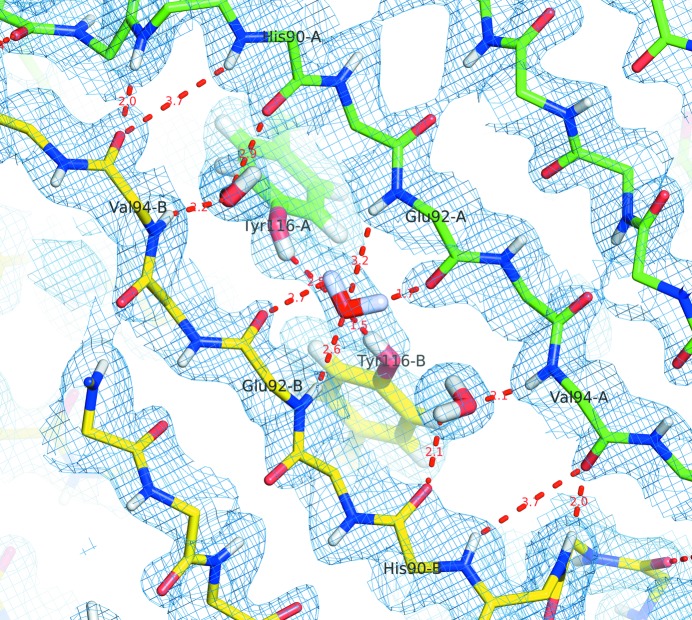
Hydrogen-bond networks in the region of the symmetry axis in the primary dimer as revealed by the 2*F*_o_ − *F*_c_ neutron map. The central water molecule is trapped between the Tyr116 η-hydroxyl groups and the Glu92 backbone carbonyl and amide groups. The water molecule establishes a very close hydrogen bond with Tyr116 in the B-chain, whereas the η-hydroxyl group of the A chain tyrosine points away from the water-oxygen atom to avoid steric hindrance.

The primary dimer relates the A-chain DAGH sheet to its B-chain counterpart H′G′A′D′, forming what appears as an almost continuous β-sheet in which the monomer–monomer interface is defined by six backbone hydrogen bonds on the binding channel side of the sheet. Fig. 3 ▶(*a*) shows full details of the close hydrogen bonding at this interface, as well as two additional hydrogen bonds involving the side-chain hydroxyl groups of residues Ser115/A with Thr119/B, and Thr119/A with Ser115/B; in all cases the neutron density maps allow direct visualization of the hydrogen atoms involved. On the other side of the monomer ‘sandwich’, as shown in Fig. 3 ▶(*b*), the A chain strands CBEF form a somewhat looser sheet-like continuation with their B-chain counterparts, F′E′B′C′. Here there are only four direct backbone hydrogen bonds joining the F strands together. The amide group of Glu89/A, at the N-terminus of strand F, interacts with the carbonyl group of Val94/B in the centre of strand F, as does the amide group of Glu89/B with the carbonyl group of Val94A. Between the space spanned by these four residues, the neutron maps show the location and orientation of three buried water molecules bridging the ND–CO contacts between the F strands. The central water molecule of the three, being in closer contact to the side-chain hydroxyl group of Tyr116/B than it is to that of Tyr116/A, highlights an important difference in the structure of the two monomers making up the primary dimer (Fig. 4 ▶).

**Figure 5:**
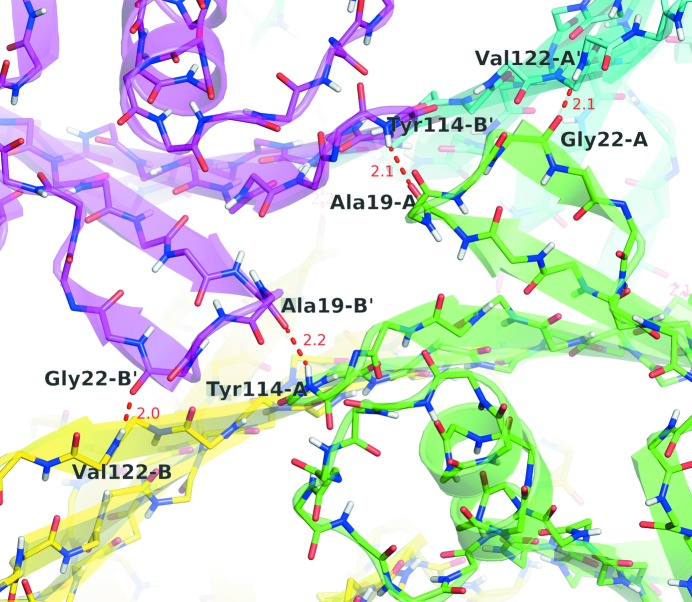
Backbone hydrogen bonds stabilizing the dimer–dimer interaction. Four out of the eight contacts are indicated with a dashed line (distances given in Å). The other four hydrogen bonds are the equivalent companions on the reverse side of the protein and are not visible in the picture.

The dimer–dimer contact is mediated by only eight backbone hydrogen bonds. The same four atoms are implicated in each monomer; Ala19/A CO interacts with the Tyr114/B′ ND, and Gly22/A CO with Val122/A′ ND, *etc.* (Fig. 5 ▶). Several residues are involved in hydrophobic contacts that further stabilize the tetramer: Val20, Leu17, Val121, Leu110 and Thr119.

The primary dimer, formed by monomers A and B, constitutes the crystallographic asymmetric unit. Some structural differences between the two monomers are clear in solvent-exposed loops (BC, CD, FG); these are likely to be imposed by the crystal lattice and would not be relevant to physiological conditions.

### 3.3. Buried water molecules   

A total of 13 buried water molecules are found per dimer. Five are located at the monomer–monomer interface. Four more are located in each of the monomers. One water molecule is situated at the junction of strands A and D, making hydrogen bonds to O/Leu12, O/Leu55 and D/Leu58. Several amyloidogenic mutations are known to be associated with these residues, with Leu55Pro being reported as the most aggressive (Sousa *et al.*, 2002 ▶).

**Figure 6:**
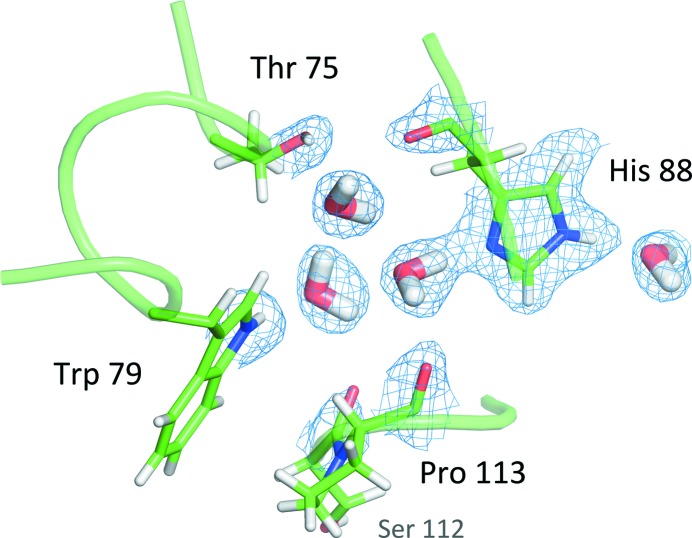
Cluster of three buried water molecules. The 2*F*_o_ − *F*_c_ neutron difference map unambiguously detects the orientation of the water molecules and the hydroxyl group of Thr75. The protonation state of His88 can be readily identified.

A group of three water molecules is trapped between the GH-loop, the residues preceding strand F, and the α-helix. The atoms involved in hydrogen bonding here are DG1/Thr75, O/His88, ND1/His88, O/Pro113, DE1/Trp79, and O/Ser112 (Fig. 6 ▶). These interactions may be important given that this is a highly conserved part of the structure, with data suggesting that only one mutation, Ser112Ile, can be tolerated. This very well ordered hydrogen-bond network is in agreement with the structure described by Yokoyama *et al.* (2012 ▶).

Another isolated buried water molecule can be found bridging the monomer–monomer interface between OG1/Thr118 from one chain and DE2/His88 from the other chain. The water molecule receives a hydrogen bond from NE2/His88 and donates a hydrogen bond to OG1/THr 118; in turn OG1 donates a hydrogen bond to the main-chain oxygen of Ala108. There are no known mutations associated with these residues. This feature is also noted in the structure reported by Yokoyama *et al.* (2012 ▶).

Three more structured water molecules are found at the monomer–monomer interface where the F-strands of chains A and B join. The residues involved here are O/His90/A, OH/Tyr116/A and D/Val94/B for the first water molecule, OH/Tyr116/A, DH/Tyr116/B and O/Glu92/A for the central water molecule, and OH/116Tyr/B, D/Val94/A and O/His90/B for the third water molecule. Mutations relating to more or less severe disease patterns are associated with all of these residues.

### 3.4. Histidine protonation states   

The four histidine residues in each of the A and B chains show different protonation states. His31 is doubly protonated in both A and B chains, as in the case in the corresponding parts of the structure reported by Yokoyama *et al.* (2012 ▶). In the case of His56, however, the neutron maps show double protonation in the A-chain residue, and single protonation (on ND1) in the B chain, whereas in the structure according to Yokoyama *et al.* there is single (NE1) protonation in both chains. For His88 there is full agreement between all structures, with single (ND1) protonation in both chains. Finally, the His90 residue is protonated on ND1 in both chains in the current structure, whereas Yokoyama and coworkers have this residue protonated on NE1. The differences noted between this structure and that of Yokoyama *et al.* may relate to the different pD values associated with the respective studies which were 6.0 and 7.4, respectively.

### 3.5. The thyroxin binding pocket   

**Figure 7:**
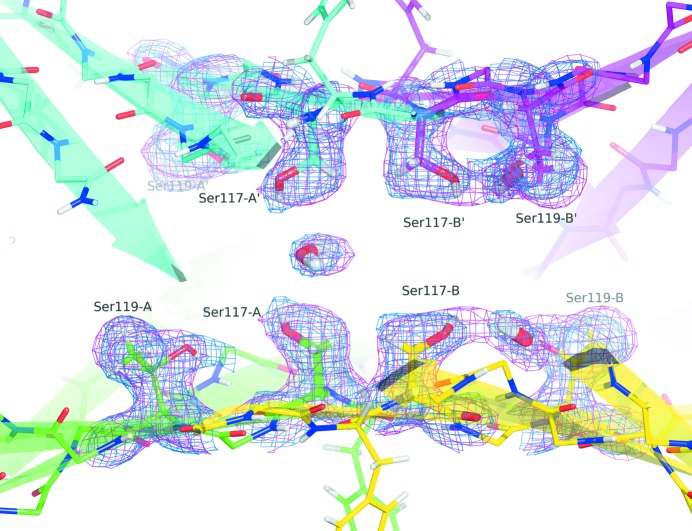
The highly symmetric centre of the homotetramer. The side chains of the four Ser117 residues form a barrier between the two hormone binding sites. The 2*F*_o_ − *F*_c_ neutron map (at 1.2 r.m.s.) shows clearly the different orientation of the γ-deuterium atoms of serine residues in chains A and B, and the presence of only one water molecule.

The intermolecular contacts formed by the dimer–dimer interface result in the formation of a spacious channel (∼40 Å long) running along the twofold symmetry axis of the protein. The channel is about 10 Å wide at the outer rim and narrows in the centre to about 4 Å. This narrowing is defined by the alignment of Ser117A and Ser117B on the bottom of the cleft, and by Ser117A′ and Ser117B′ on the top (Fig. 7 ▶), accommodating two separate binding sites. Small, though important, differences between subunits A and B result in different geometries for the two hormone binding sites. Monomers A and A′ form site A, and monomers B and B′ form site B, each binding pocket being internally symmetric due to the twofold symmetry axis that runs through the central channel of the tetramer. The central part of this channel, close to Ser117, is quite similar for both binding sites, although the protons of the Ser117 hydroxyl groups have different orientations in the A and B monomers (Fig. 7 ▶). In the A site, the γ-hydrogen atoms chelate a structured water molecule, whereas in site B the γ-hydrogen atoms are rotated by about 20° thus resulting in a 0.5 Å widening of the pocket (distance DG–DG′ 4.1 Å in the A site *versus* 4.7 Å in the B site); the neutron data show no bound water at the B site. This important detail cannot be derived from X-ray models with riding hydrogen atoms and is consistent with the neutron data published by Yokoyama *et al.* (2012 ▶). The two binding pockets show subtle structural differences. The charged amino acids Lys15 and Glu54 that define the position of thyroxine adopt very similar positions and are well structured with low *B*-factors. However their hydration states are different. The larger binding pocket B, which has been postulated to have the higher binding affinity, has a well defined water molecule bound to Lys15, whereas in the narrower site A no hydration is noted.

## 4. Discussion   

Despite its ability to form fibrils, transthyretin is a very stable protein. It withstands temperature changes from 277 to 353 K and pH variation between 5.5 and 12.0 without significant damage (Lundberg *et al.*, 2009 ▶). Studies concerning its evolutionary development show that the central residues in the binding channel have not changed in over 400 million years (Prapunpoj & Leelawatwattana, 2009 ▶). The tertiary structure shows an equally stable architecture that cannot easily be disrupted. A comparison of 23 structures of wild-type and mutant TTR showed no evidence for significant structural changes between amyloidogenic, non-amyloidogenic or wild-type TTR quarternary structure (Hörnberg *et al.*, 2000 ▶). In fact, the authors of this study concluded that there was greater structural variability amongst published X-ray structures of wild-type TTRs originating from different research groups. However, the fact that most of the known mutations in the TTR gene have important implications for amyloidosis suggests that there are aspects of TTR structure that are not fully understood. We believe that the results described here, particularly those relating to the hydration and protonation states of the protein, may provide a fuller understanding of the factors important in TTR stability and disassembly.

### 4.1. Ser117 – the pivotal point   

A common characteristic of vertebrate TTR, with the exception of fish, is the occurrence of the four Ser117 residues in the centre of the molecule (Prapunpoj & Leelawatwattana, 2009 ▶). Ser117 has been reported as capable of subtle conformational changes upon ligand binding (Morais-de-Sá *et al.*, 2004 ▶). This residue is located at the centre of the tetramer, where the crystallographic (dimer–dimer interface) and noncrystallographic (monomer–monomer interface) sym­metry axes intersect. A comparison of several X-ray structures from the PDB shows considerable variation in the assigned water structure in the binding channel – ranging from zero to four water molecules situated between the central serine residues, and it is therefore hard to be sure of the significance of these observations. In this study, both of our independent neutron datasets, which reveal water molecules in greater detail than corresponding X-ray analyses, show the presence of a single highly ordered water molecule, coordinated by Ser117A and Ser117A′. This water molecule mediates an important contact on one side of the dimer–dimer interface (Fig. 7 ▶). We note this to be consistent with the analysis of Yokoyama *et al.* (2012 ▶). The neutron data also show the hydroxyl group orientation that stabilizes this water interaction with good hydrogen bonding geometry.

### 4.2. Implications for the thyroxine binding cleft   

The location of the Ser117A/Ser117A′ hydroxyl-water interaction highlights the importance of subtle structural differences between the A and B chains, as well as the fact that the two thyroxine binding sites are not equivalent (see Fig. 7 ▶). Early binding studies on human TTR with thyroxine suggested a negative binding cooperativity, supported by a decrease in the binding constant for the second thyroxine by a factor of 100; the corresponding *K*_1_ and *K*_2_ dissociation constants are approximately 10 n*M* and 1 µ*M*, respectively (Ferguson *et al.*, 1975 ▶). Other monovalent binders such as diflunisal and its analogues only occupy on average one binding site (Adamski-Werner *et al.*, 2004 ▶; Castaño *et al.*, 2012 ▶). However, it is hard to determine the ratio of unbound, singly bound or doubly bound TTR tetramer (Wiseman *et al.*, 2005 ▶). Only bivalent compounds force the mutual occupation of both binding sites in the protein, although they are spatially demanding and imply a partial dissociation of the tetramer before they can occupy the entire channel (Green *et al.*, 2003 ▶). Following this, other homo-bivalent inhibitors have been designed to cross link the tetramers and thus speed up the clearance of TTR from serum. Furthermore, under physio­logical conditions, these constructs bind to wtTTR and show an increased binding strength and stabilization of the tetramer (Kolstoe *et al.*, 2010 ▶).

**Figure 8:**
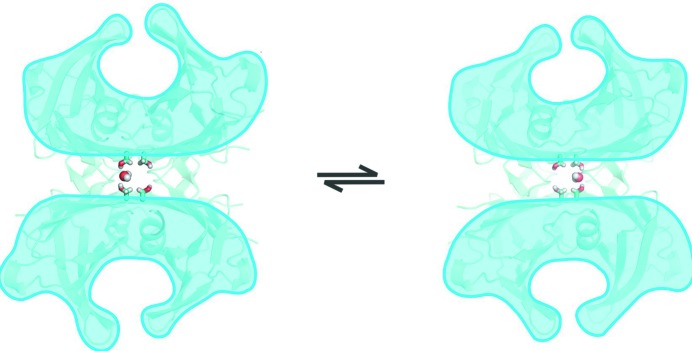
Schematic illustration showing the proposed solution equilibrium between the two putative conformations of the A and B binding pocket. The entry to the binding pocket is wider for the B than for the A pocket as a result of the water molecule complexed between the Ser117 residues of monomers A and A′. This draws together the dimers, and thereby narrows the A binding pocket.

This neutron analysis shows an asymmetry at the dimer–dimer interface, leading to different opening angles of the channels. In solution the protein is very likely to flex back and forth, interchanging the two water molecules bound by the Ser117 between the A–A′ site and the B–B′ site, thereby slightly opening one or the other binding pockets. It has been suggested that the slightly larger binding site is also the one with the higher binding affinity (Neumann *et al.*, 2001 ▶). Immobilized in the crystal lattice, the existence of two different ground states becomes apparent. Based on this evidence, our structure supports the concept of two distinct asymmetric binding sites having different affinities (Fig. 8 ▶). Binding of the first ligand in the slightly bigger site will restrain the protein’s flexibility, so that only the lower affinity binding pocket is available. This assumption is supported by the observation that the structure does not significantly change upon ligand binding (Hörnberg *et al.*, 2000 ▶).

### 4.3. From the native fold to fibrils – a ‘dimer-only’ hypothesis   

There are numerous theories concerning the fibril formation of transthyretin. One of the most popular is the hypothesis that a complete degradation to the monomeric species has to occur prior to aggregation (Lundberg *et al.*, 2009 ▶; Wiseman *et al.*, 2005 ▶). Other publications suggest a mechanism *via* the dimeric state of the protein (Serag *et al.*, 2001 ▶). This model is supported by the findings that there is a concentration of amyloidogenic mutations in the edge strands C and D, whereas there are few at the dimerization interfaces (Serpell *et al.*, 1996 ▶). A model for amyloid formation originating from the tetrameric state has also been proposed (Eneqvist *et al.*, 2000 ▶). There is however broad consensus that the resulting amyloid filament is a cross-β structure having ∼4.7 Å inter-strand spacing and ∼10.4 Å inter-sheet spacing (Blake & Serpell, 1996 ▶).

The idea that amyloid formation occurs from dissociated monomers is at variance with the fact that even for mutated transthyretin, harsh denaturing conditions (*e.g.* acidic pH close to the pI) have to be deployed to disrupt the tetrameric structure of the protein and generate fibrils **in vitro**. The fibrils so formed are usually of poor quality and do not (as visualized by electron microscopy) resemble fibrils from natural sources. Furthermore, TTR disassembles into a dimer when exposed to 5% SDS, and the monomeric form is only obtained after boiling the sample (Lundberg *et al.*, 2009 ▶). A probe for the existence of a monomeric species **in vivo** is the measurement of subunit exchange under physiological conditions. By mixing tagged wtTTR with non-tagged protein it has been shown that subunit exchange can be observed after 40 h, and equilibrium is reached after seven days (Schneider *et al.*, 2001 ▶). However, in nature the half-life of TTR in the blood is less than two days (Ingenbleek & Young, 2002 ▶). This rather short biological half-life ensures rapid disposal of denatured protein prone to form fibrils, although some TTR clearly circumvents the pathway.

At the monomer–monomer interface, 12 inter-monomer hydrogen bonds and about seven intermolecular hydrophobic interactions (π-stacking, hydrophobic side-chain arrangements) were identified. The interactions on the dimer–dimer interface are much weaker. For a surface that is more than twice as big, only eight inter-dimer hydrogen bonds can be allocated and few unspecific hydrophobic contacts. Thus, it is very likely that the dimer–dimer dissociation event prevails over monomer–monomer dissociation. Stereochemically, it is also easy to see how the β-sheet may be continued *via* a simple dimerization of the edge-strands C and D (residues 45–58) with the corresponding strands from another dimer. Only minor structural rearrangements are necessary to better expose the amide and carbonyl groups to allow this association with a binding partner. Once the first building block has formed, it is only a matter of time and protein supply to sustain fibril formation.

The slow build-up rate of fibrils due to the necessity of nucleation presumably results in an exponential growth rate, with a variable early age onset for mutated protein *versus* a late onset for wtTTR.

## 5. Conclusions   

Hydrogen accounts for almost 50% of all atoms present in a protein. Neutron studies such as this one provide a unique opportunity to reliably image these important atoms, and the use of perdeuterated TTR has proved to be crucial. The data presented here emphasize the importance of determining the hydrogen-atom positions directly rather than assuming them on the basis of chemical context. For example, the fact that the hydrogen atom of the γ-hydroxyl group of Ser117A is rotated by about 20° compared with the same residue on the B-chain cannot be predicted on the basis of the very small conformational change of the oxygen atom of the same group. This subtle difference affects the quaternary protein structure at the dimer–dimer interface, resulting in two hormone binding sites with distinct binding constants.

The information on the location and orientation of buried water molecules provides insight to the specific parts of TTR that are believed to be important in stabilizing the structure of the primary dimer. This is significant for developing a structural basis for understanding mutations of medical significance and that are known to enhance fibril formation. The ordered water structures around His88, Thr75, Trp79 and Ser112, are therefore of particular significance, as is the protonation state of His88. Neutron crystal structures of key mutants would therefore be of clear benefit to this field.

The hydrogen-bonding network connections identified here corroborate the hypothesis that a dimer is the principal building block of TTR amyloid fibrils and that a complete dissociation into monomers prior to fibril formation is not likely. We are confident that these findings will be helpful for a better understanding of the properties and dynamics of wild-type transthyretin, and may lead to exploitable information to counter amyloidosis.

## Supplementary Material

PDB reference: X-ray structure of human transthyretin to 1.9 Å resolution, 4pvl

PDB reference: neutron structure of human transthyretin to 2.0 Å (Laue), 4pvm

PDB reference: neutron structure of human transthyretin to 2.3 Å (monochromatic), 4pvn

## Figures and Tables

**Table 1:** Summary of data-collection and analysis statistics

	Laue neutron diffraction	Monochromatic neutron diffraction	X-ray diffraction
Data collection			
Beamline	LADI-III at ILL	D19 at ILL	ID23-1 at ESRF
Temperature (K)	293	293	293
Wavelength ()	3.244.18	2.42	0.9791
Resolution ()	43.682.00 (2.112.00)	52.212.30 (2.422.30)	52.281.85 (1.951.85)
No. reflections measured	69034 (6128)	40015 (4199)	76578 (10682)
No. unique reflections	13480 (1524)	11358 (1613)	21718 (3060)
Completeness (%)	78.3 (62.1)	99.3 (98.7)	99.4 (97.9)
No. of images	23	N/A	180
Oscillation angle ()	Step angle = 7	Scan step 0.07	0.5
Exposure time per image (s)	18470 (5.13hours per image)	87 or 173	0.1 (beam attenuated to 0.74%)
Space group	*P*2_1_2_1_2	*P*2_1_2_1_2	*P*2_1_2_1_2
Unit-cell parameters ()†	*a* = 43.68, *b* = 86.26, *c* = 65.72	*a* = 43.68, *b* = 86.26, *c* = 65.72	*a* = 43.68, *b* = 86.26, *c* = 65.72
*R*_merge_	0.182 (0.337)	0.152 (0.645)	0.093 (0.420)
*R*_p.i.m._	0.078 (0.162)	0.096 (0.470)	0.057 (0.254)
Multiplicity	5.1 (4.0)	3.5 (2.6)	3.5 (3.5)
Mean *I*/(*I*)	7.3 (3.4)	7.7 (1.6)	7.4 (2.6)
Wilson *B* factor (^2^)	15.7	14.9	19.8
Refinement	Joint NX	Joint NX	X only
Resolution range ()	N: 36.382.00 (2.072.00)	N: 52.282.30 (2.532.30)	43.131.95 (2.001.95)
	X: 43.131.95 (2.001.95)	X: 43.131.95 (2.051.95)	
PDB code	4pvm	4pvn	4pvl
No. reflections N	13391 (923)	11395 (2630)	
No. reflections X	18655 (1274)	18655 (2512)	18656 (1274)
Completeness (%) N	76.9 (60.0)	98.6 (98.0)	
Completeness (%) X	99.4 (100.0)	99.4 (100.0)	99.5 (100.0)
*R*_work_ N	0.209 (0.280)	0.209 (0.258)	
*R*_work_ X	0.153 (0.173)	0.156 (0.185)	0.152 (0.175)
*R*_free_ N	0.271 (0.320)	0.262 (0.322)	
*R*_free_ X	0.203 (0.220)	0.185 (0.226)	0.201 (0.198)
No. protein atoms	3630	3680	1815 (no H or D)
No. main-chain amide-H atoms with H_occ_ > 0.5	29	34	0
No. D_2_O, DO, O-only molecules	36, 12, 41	30, 18, 36	148
R.m.s.d. bond length ()	0.013	0.012	0.011
R.m.s.d. bond angles ()	1.354	1.309	1.282
Average *B* factors (^2^)			
Main chain (A/B)	18.5/23.4	23.1/29.1	20.7/25.4
Side chain (A/B)	23.7/28.6	29.3/35.7	28.7/33.2
Solvent	26.8	46.4	40.9
Ramachandran statistics (%)			
Favoured	98.3	98.3	97.8
Allowed	1.3	1.3	2.2

† X-ray cell dimensions were used for both neutron structures.
